# Identification of Yellow Pigmentation Genes in *Brassica rapa* ssp. *pekinensis* Using Br300 Microarray

**DOI:** 10.1155/2014/204969

**Published:** 2014-12-31

**Authors:** Hee-Jeong Jung, Ranjith Kumar Manoharan, Jong-In Park, Mi-Young Chung, Jeongyeo Lee, Yong-Pyo Lim, Yoonkang Hur, Ill-Sup Nou

**Affiliations:** ^1^Department of Horticulture, Sunchon National University, Suncheon, Jeonnam 540-950, Republic of Korea; ^2^Department of Agricultural Education, Sunchon National University, Suncheon, Jeonnam 540-950, Republic of Korea; ^3^Plant Systems Engineering Research Center, KRIBB, Daejeon 305-806, Republic of Korea; ^4^Department of Horticulture, Chungnam National University, Daejeon 305-764, Republic of Korea; ^5^Department of Biological Sciences, Chungnam National University, Daejeon 305-764, Republic of Korea

## Abstract

The yellow color of inner leaves in Chinese cabbage depends on its lutein and carotene content. To identify responsible genes for yellow pigmentation in leaves, the transcriptome profiles of white (Kenshin) and yellow leaves (Wheessen) were examined using the Br300K oligomeric chip in Chinese cabbage. In yellow leaves, genes involved in carotene synthesis (*BrPSY, BrPDS, BrCRTISO*, and *BrLCYE*), lutein, and zeaxanthin synthesis (*BrCYP97A3* and *BrHYDB*) were upregulated, while those associated with carotene degradation (*BrNCED3, BrNCED4*, and *BrNCED6*) were downregulated. These expression patterns might support that the content of both lutein and total carotenoid was much higher in the yellow leaves than that in the white leaves. These results indicate that the yellow leaves accumulate high levels of both lutein and *β*-carotene due to stimulation of synthesis and that the degradation rate is inhibited. A large number of responsible genes as novel genes were specifically expressed in yellow inner leaves, suggesting the possible involvement in pigment synthesis. Finally, we identified three transcription factors (*BrA20/AN1-like, BrBIM1*, and *BrZFP8*) that are specifically expressed and confirmed their relatedness in carotenoid synthesis from *Arabidopsis* plants.

## 1. Introduction

Chinese cabbage (*Brassica rapa* ssp.* pekinensis*), which is widely used vegetable in Asia, particularly in China, Japan, and Korea, is rich in vitamin C and minerals such as potassium, calcium, magnesium, and zinc. This subspecies is typical of the* Brassica* A genome and has a large number of genomic resources including a mapping population and BAC libraries, due to its small genome (529 Mb) relative to other* Brassica* species. The inner leaves can be white, yellow, or orange. Earlier reports have revealed that the color of the inner leaves is primarily associated with their carotenoid composition. Yellow inner leaves of Chinese cabbage have higher nutritional values than white inner leaves [1]. In recent years, Asian countries have preferred yellow inner leaf cultivars due to its high nutritional values and significant economic benefits.

Carotenoids represent a class of red, orange, and yellow pigments widely distributed in nature that are mainly C_40_ isoprenoids and play a critical role in human nutrition and health. Because humans are unable to synthesize vitamin A* de novo* from endogenous isoprenoids precursors, it is primarily obtained through dietary plant carotenoids. Apart from their nutritional significance, carotenoids have been implicated in reducing the risk of cancer and cardiovascular diseases via their antioxidant activity [2, 3]. Thus, development of carotenoid enriched food crops provides the most effective and sustainable approach to maximizing the nutritional and health benefits of carotenoids to a large number of populations worldwide.

The carotenoid pigment composition has been investigated in many Brassica vegetables including broccoli, Brussels sprouts, white cabbage, red cabbage, kale, and cauliflower [4] and these investigations have indicated that lutein and *β*-carotene were the dominant carotenoids. Chinese cabbage also contains lutein and *β*-carotene as the major carotenoid pigment [5], with levels of 0.01 to 0.03 mg/100 g fresh weight generally being observed [6]. The orange-yellow pigmentation of Chinese cabbage expressed in inner leaves and petals is governed by a single recessive gene. The locus of the pigmentation has been mapped from three RFLP markers closely linked to each other [7]. Microarray technology enables genome wide analyses that can be used to identify plant gene expression under various conditions, including plant organogenesis and interactions with microorganisms [8, 9]. Microarray experiments have been employed to analyze gene expression changes in a number of crop species, including Chinese cabbage [10, 11].

The objective of this study was to identify the genes responsible for yellow color pigmentation in* B. rapa* inner leaves using a 300K* Brassica rapa* microarray with 47,548 unigenes.

## 2. Materials and Methods

### 2.1. Plant Materials

Two lines of Chinese cabbage (*Brassica rapa* ssp.* pekinensis*) were used in this study: hybrid cultivar Wheessen (Woori Seed Co. Korea) for yellow inner leaves and the inbred line Kenshin for white inner leaves (Figure S1 in Supplementary Material available online at http://dx.doi.org/10.1155/2014/204969). Seedlings were transplanted to cabbage patch at Chungnam National University, Daejeon, Korea, on September 1. After completion of the heading process (November 1), inner leaves were harvested from 10 plants. The blade portion of the leaves (excluding the midrib) was separated, frozen in liquid nitrogen, and stored at −70°C until use.

The wild-type strain and T-DNA insertional mutants (SALK_045674, SALK_008677C, SALK_098442C, and SALK_012835) of* Arabidopsis thaliana* (L.) Heynh var. Columbia (Col-0) were collected from ABRC (http://www.arabidopsis.org/). Seeds were sterilized with 50% bleach solution with 0.1% triton X-100 (Sigma, USA), followed by germination on 60 mm × 60 mm pots in potting soil. Plants were grown in growth chamber at 22 ± 0.5°C, with a photon flux density of 140 ± 2 *μ*mol/m^2^/sec and for a 16 h light/8 h dark photoperiod. Mutants were confirmed by PCR using primers that were specific for the gene sequence and the T-DNA left border (Table S1). After 3 weeks, all leaves from 20 plants were collected and subjected to measure the carotenoid content.

### 2.2. Measurement of Lutein and Carotene Content from Chinese Cabbage

Leaf samples from each plant were ground in liquid nitrogen using a mortar and pestle after which 1 mL DMSO and 10 mL methanol were added to 1 g of sample and mixed well. The sample was then incubated in darkness for 30 min after which 5 mL of hexane and 10 mL of 20% NaCl were added. Next, samples were centrifuged at 2100 ×g for 10 mins, after which the supernatant was collected and amended with 0.5 g of sodium sulfate. The samples were then vortex and centrifuged at 2100 ×g for 10 min. Finally, the supernatant was filtered through a 0.45 *μ*m PTFE and analyzed by high performance liquid chromatography (HPLC).

The lutein and carotene content were analyzed using an HPLC (Waters Corp., Milford, Mass., USA) equipped with a 2707 autosampler, a 1525 binary pump, and a C18 reversed-phase symmetry analytical column (2.6 *μ*m × 100 mm × 4.6 mm) with a guard column containing the same packing material as the analytical column (Phenomenex Kinetex). The following binary mobile phases were used during analysis: solvent A, 75% methanol in HPLC grade water; solvent B, ethyl acetate. The gradient for the HPLC analysis was linearly applied as follows: 0% B at zero min, 75% B at 10 min, and 100% B at 14 min. Flow rate was set to 1.0 mL/min at constant room temperature, the Waters UV/visible detector was set at 450 nm and carotenoid standards [lutein xanthophyll (alfalfa sigma) and *β*-carotene (wako)] were used to generate characteristic UV visible spectra and calibration curves.

Individual carotenoids in the samples were tentatively identified based on comparison of their individual UV-visible spectrum and retention time. Lutein and *β*-carotene content were analyzed for each sample on the day of analysis. The percentage of lutein and *β*-carotene was calculated per 1 g of raw sample.

### 2.3. Measurement of Total Carotenoid Content from* Arabidopsis* Plants

The content of total carotenoids from* Arabidopsis* plants was assayed by a simple and efficient method by Porra et al. [12]. Leaves were ground in liquid nitrogen with mortar and pestle. One mL of DMF (dimethylformamide) was added to ca. 100 mg of powder in the dark. The suspension was microfuged by 12,000 rpm at 4°C for 10 min and supernatant was diluted by 10 times with DMF. The absorbance of the resulting solution was measured at 461 and 664 nm. The carotenoid content (*μ*g/mL) was calculated by an equation: [A_461_ − (0.046 × A_664_)] × 4. Finally, its value was transformed into *μ*g/g tissue.

### 2.4. Construction of the Br300K Oligomeric Chip and Microarray Analysis

A 300k microarray chip (Br50K; version 2.0) for* B. rapa* designed from 47,548 unigenes (Table S2) was manufactured by NimbleGen, Inc. (http://www.nimblegen.com/) as recently described [13]. To assess the reproducibility of the microarray analysis, we repeated the experiment two times using independently prepared total RNA. The data were then normalized and processed with cubic spline normalization using quantile to adjust signal variations between chips and robust multichip analysis (RMA) using a median polish algorithm implemented in NimbleScan [14, 15]. RNA preparation, GeneChip hybridization, and data analyses were also conducted following previously described methods [16].

### 2.5. RT-PCR Analysis

Total RNA (5 *μ*g) from each sample was added with random hexamer primers in a superscript first-strand cDNA synthesis system according to the manufacturer's instructions (Invitrogen, USA). Complementary DNA was diluted 10-fold and 1 *μ*L of the diluted cDNA was used in a 20 *μ*L PCR mixture. RT-PCR primers are listed in Table S1 and the primers for* BrACT1,* which was used as a control, were 5′-AGATCGTCCCCGGCTTCAAA-3′ (forward) and 5′-CAAGGCGTAAACGACGCAGG-3′ (reversed). Standard PCR was performed by subjecting the samples to 5 min initial denaturation at 94°C, followed by 25 cycles of 94°C for 30 s, 55°C for 30 s, and 72°C for 90 s. PCR products were electrophoresed on a 1% agarose gel.

## 3. Results 

### 3.1. Carotenoid Analysis

We compared the carotenoid composition including lutein and *β*-carotene in the inner leaves of Wheessen (yellow inner leaves) with those of Kenshin (white inner leaves) using HPLC analysis. The content of lutein and *β*-carotene is listed in Table 1. A lutein-representing peak was present in both Wheessen and Kenshin, although Wheessen showed a peak area higher than that found in Kenshin (data not shown). The content of lutein and *β*-carotene in Wheessen inner leaves was higher than that of Kenshin inner leaves by 250- and 17-fold, respectively. These data were consistent with those observed for standard lutein and *β*-carotene. The results supported that carotenoid becomes the main pigment in inner leaves of Chinese cabbage and is responsible for their yellow color.

### 3.2. Microarray Analysis

To evaluate the pigmentation of yellow inner leaves in Chinese cabbage, we conducted microarray analyses using the version 2 Br300K chip (Table S2). Among 47,548 genes on the Br300K chip, 11,543 genes showed probe intensity (PI) values of less than 500 from two tested leaf samples. To reduce the false positives, we ignored these 11,543 genes in subsequent analyses, while the remaining 36,005 genes were subjected to significance analysis of microarray (SAM) [17]. Thus, genes with adj.*P*.Value or false (FDR) discovery rate below 0.05 were collected and further selected for those genes with expression greater than 2-fold or less than −2-folds. A total of 10,199 were differentially expressed: 4,807 that were upregulated and 5,392 that were downregulated in yellow inner leaves (Table S3). Highly expressed genes in yellow leaves, like the top 20 upregulated genes in yellow inner leaves (Tables S3 and S4), appeared to be new genes whose functions have not been identified in plants up to now. Some of these novel genes might be directly or indirectly involved in the determination of yellow color in Chinese cabbage.

Interestingly, Br300K microarray included 8,542 unigenes classified as no hit found upon initial analysis with* Arabidopsis thaliana* annotation. Among these, 180 unigenes were specifically expressed in yellow leaves (Table S5). When these sequences were subjected to BlastN analysis, some of the unigenes showed low similarity to* Brassica* sp. and other plant unigenes without known domain up to now (data not shown). Identification of function of these genes will be indispensable for further researches if the efficient and fast method of* B. rapa* transformation is available.

### 3.3. Functional Categorization of Genes

**Figure 1:**
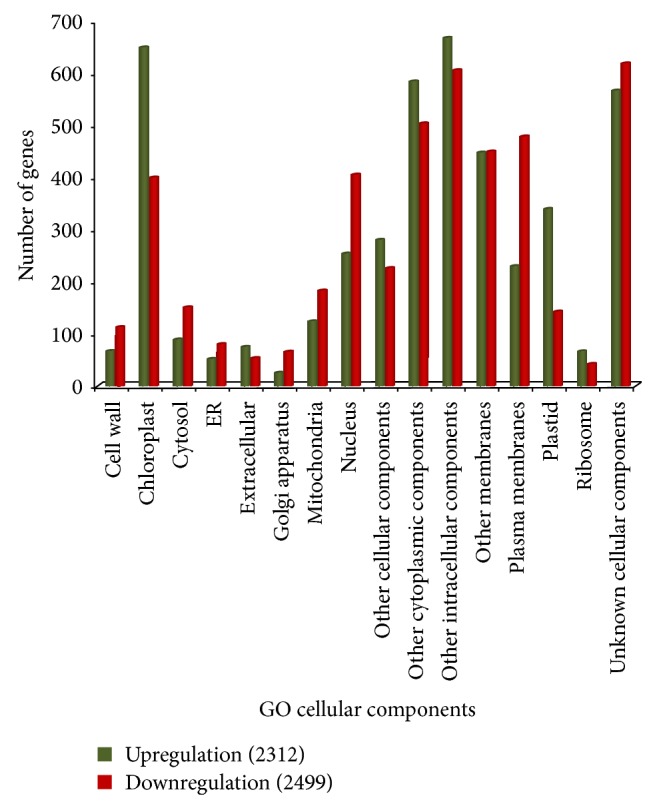
Gene ontology characterization by loci for cellular components.

**Figure 2:**
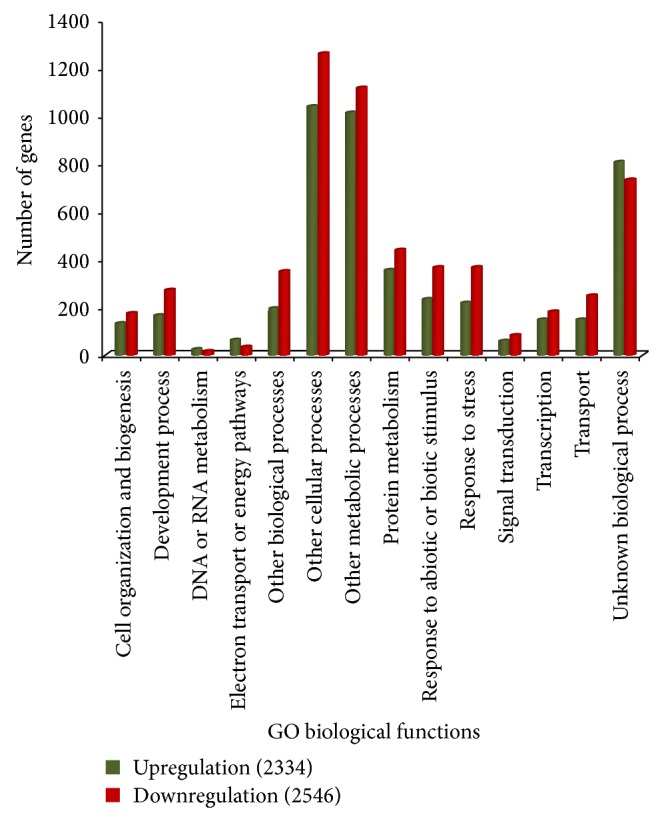
Gene ontology characterization by loci for biological functions.

**Figure 3:**
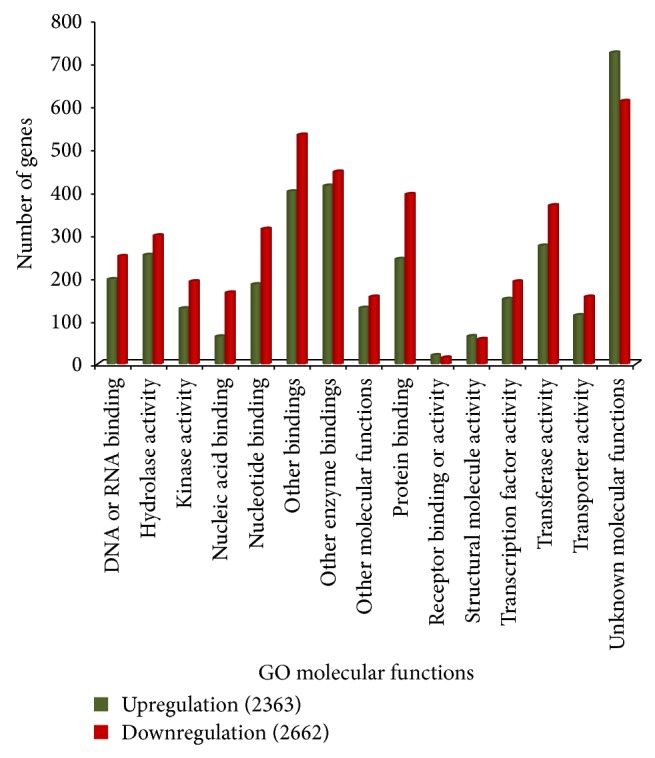
Gene ontology characterization by loci for molecular functions.

Gene functions were analyzed based on gene ontology (GO) to simplify the annotation process using controlled vocabularies and hierarchies including three major categories, cellular component (CC), biological process (BP), and molecular function (MF) [18]. All EST sequences that showed greater than 2-fold increases were classified into 2,312, 2,334, and 2,546 ESTs of CC, BP, and MF, respectively, while there were 2,499, 2,546, and 2,662 downregulated genes of CC, BP, and MF, respectively. The majority of the differently expressed genes were shown to be involved in metabolic and cellular process, and some were components of the plasma membrane and plastids (chloroplast). The unique sequences were grouped under the first category of CC (Figure 1), which contained cell wall, chloroplast, cytosol, ER, extracellular region, Golgi apparatus, mitochondria, nucleus, other cellular components, other cytoplasmic components, other intracellular components, other membranes, plasma membranes, plastid, ribosome, and unknown cellular components. Previous studies have reported that carotenoids are also synthesized and accumulated in lipid bodies inside the chromoplasts and plastids which accumulate pigments in flowers, fruits, and storage roots. Carotenoids are more stable in chromoplasts than in plastids because they are protected from light [19]. The second category included BP (Figure 2), with subcategories such as cell organization and biogenesis, development processes, DNA or RNA metabolism, electron transport or energy pathways, other biological processes, cellular processes, metabolic processes, protein metabolism, response to abiotic or biotic stimulus, stress, signal transduction, transcription, transport, and unknown biological processes. Although enzymes appear to be of particular importance during biological process responses, some carotenogenic enzymes such as geranylgeranyl diphosphate (GGDP) synthase (GGPS) and phytoene synthase (PSY) from the isoprenoid pathway are part of a soluble large protein complex that catalyzes the formation of phytoene in plastid stroma [20]. A large protein complex PDS catalyzes the synthesis of *α*- and *β*-carotene from phytoene in the plant plastid membrane [21]. This phytoene enzyme plays a key role in the carotenoid biosynthetic pathway. The large numbers of unique sequences were grouped under the third category of MF (Figure 3), with DNA or RNA binding, hydrolase activity, kinase activity, nucleic acid binding, nucleotide binding, other binding, other enzyme binding, other molecular functions, protein binding, receptor binding or activity, structural molecule activity, transcription factor activity, transferase activity, transporter activity, and unknown molecular functions. The expression of many genes targeted to plastids is regulated through a signal between the nucleus and the plastids. These signals, whether environmental or developmental, are supposed to include reactive oxygen species (ROS), carotenoids, and hormones. Thus, plastids seem to be important to carotenoid biosynthesis [22].

### 3.4. Expression of Lutein and *β*-Carotene Synthesis-Related Genes

To understand the relatedness of known genes that regulate the carotenoid level, expression of several categories of* B. rapa* (*Br*) orthologous genes was analyzed (Table 2). These genes include *α*- and *β*-carotene synthesis-related genes (phytoene synthase (*PSY*),* PDS*, carotene isomerase (*CRTISO*), and lycopene epsilon cylase (*LUT2*)), lutein and zeaxanthin synthesis-related genes (carotene epsilon-monooxygenase (*RUT1*), lutein deficient 5 (*LUT5*), carotene *β*-hydroxylase 1 (*HYDB1*), and* HYDB2*), and carotenoid degradation genes (9-cis-epoxycarotenoid dioxygenase 3 (*NCED3*),* NCED4,* and* NCED6*). Three known color-related genes (*pale CRESS1* (*PAC1*), cauliflower orange (*OR*), and* immutans* (*IM*)) were used for references. As shown in Table 2, the expression of genes associated with carotenoid synthesis was higher in yellow inner leaves compared to that in white inner leaves, whereas the expression of degradation related genes was lower. The level of* Or* gene that controls *β*-carotene content [23] was similar in both inner leaves, while* PAC1* level that controls early chloroplast development [24] was high in white leaves, suggesting these genes may not be related to the determination of yellow color. However, the level of* IM* which determines source-sink interactions which appeared to be little higher in yellow leaves rather than white inner leaves, suggesting somewhat relatedness of chloroplast development with carotenoid biosynthesis. Microarray analysis was conducted in duplicate using biological repeated samples and correlation coefficient between replicated microarray analyses was found to be greater more than 0.96 (Figure S2). The RT-PCR expression ratio was compared with our microarray results and correlation coefficient values were found to be greater than 0.8 (Figure S3). Unfortunately, some RT-PCR results were not consistent with PI values, suggesting requirement of real-time PCR experiment.

### 3.5. Identification of Yellow-Specific Responsive Genes

In addition to carotenoid biosynthetic genes, some of genes that are predominantly expressed in yellow inner leaves will be either directly or indirectly related to the yellow color determination in Chinese cabbage. To identify leaf-color specific genes, we selected genes as follows. Yellow-specific genes were defined as those that had PI values of over 1000 in yellow leaves but less than 500 in white leaves (Table S6), while white specific genes were those that had PI values of over 1000 in white leaves but less than 500 in yellow leaves (Table S7). The total numbers of yellow and white specific genes were 761 and 647, respectively, implying that the expression of a large number of genes is essential for determination of pigmentation and other traits in each line. These inner color-specific genes also include many unidentified genes up to now. We confirmed expression of some of color-specific genes by RT-PCR (Table 3). As shown in Table 3, many genes were unidentified genes or* B. rapa* specific genes. In addition, some genes, like chitinase and taumatin protein gene, might not be related to leaf color determination. These results indicate that many of color-specifically expressed genes could be due to cultivar traits, while some of them might be related to color determination.

### 3.6. Identification of Transcription Factor Genes Induced Specifically in Yellow Leaves

Transcription factors control the expression of a genome and play vital roles in the plant life cycle. Therefore, we examined the transcription factors associated with yellow pigmentation [25]. Among 2,336 transcription factor genes, 536 clones were found to be expressed in low levels with a PI value < 500 for both yellow and white leaves, while 312 clones were constitutively expressed in both leaves (Table S8). Twenty-one transcription factors responsive to yellow color in* B. rapa* were identified by comparison with* Arabidopsis* information (Table 4). Although these TFs have not been reported for their function with respect to carotenoid biosynthesis yet, there is a possibility of involvement of these genes in color determination in* B. rapa*, directly or indirectly. Particularly, three genes showing high levels of expression in yellow inner leaves will be good candidates for yellow color regulation. These include* Brapa_ESTC013161* (*A20/AN1-like zinc finger protein*),* Brapa_ESTC025847* (*BIM1* (BES1-interacting MYC-like protein 1)), and* Brapa_ESTC006452* (*ZFP8* (*Zinc finger protein8*)). These genes may be responsible for the carotenoid synthesis or related to pigment metabolism during carotenoid production.

Four T-DNA knock-out* Arabidopsis* mutants corresponding to three* B. rapa* genes (*Brapa_ESTC013161*,* Brapa_ESTC025847*, and* Brapa_ESTC006452*) were analyzed for carotenoid content by comparison with* Arabidopsis* wild type (Table S9). The content of total carotenoid was greatly reduced in T-DNA knock-out mutants, implying their possible function in the carotenoid biosynthesis. Particularly knock-out of* A20/AN1-like zinc finger protein* gene greatly affected the carotenoid content.

## 4. Discussion

Transcriptome studies using microarray analyses are increasingly being used to identify key genes involved in plant growth, development, and responses to environmental changes. Nevertheless, microarray technology has not been widely applied to the study of* Brassica* species owing to the absence of a high density* Brassica* microarray with sufficient coverage of the entire genome. Instead,* Arabidopsis* microarray has been widely employed for analysis of* Brassica* species because both species belong to the same family (Brassicaceae) and have an evolutionary close relationship. The* Brassica* genome has generally diverged from* Arabidopsis* [26, 27] and consists of approximately 46,000 genes [28]. Although the* Arabidopsis* microarray chip may provide some information regarding gene regulation of* Brassica* plants, it is not sufficient to provide information regarding* Brassica* biology [29]. Therefore, to accurately conduct transcriptome studies, a* Brassica* specific microarray Br300K was used in the present study. This microarray will also be useful in investigations of other* Brassica* species, including* B. napus* and* B. oleracea*.

Carotene and lutein contents are known key factors associated with yellow pigmentation in Chinese cabbage. However, no reports of specific genes involved in yellow color determination in Chinese cabbage have been published to date, except the involvement of* BrCRTISO* in orange color determination [30]. Researches to identify genes regulating yellow inner leaves and to confirm inheritance of its characteristics in Chinese cabbage are essential for the improvement of Chinese cabbage quality. We identified many genes that were specifically expressed in yellow inner leaves using microarray experiment and confirmed some of them by RT-PCR. Furthermore, a high correlation was observed between microarray data of the two samples. In addition, a high correlation between RT-PCR analysis and microarray data indicated that our data from the Br300K microarray were reliable and reproducible.

Wheessen and Kenshin were used for HPLC analysis to compare carotenoid composition. In a previous study, lutein was found to be predominantly accumulated in yellow inner leaves [5, 31]. Carotenoid composition, especially lutein in the inner leaves of yellow cultivar, was higher than that in the inner leaves of orange cultivar upon HPLC analysis [30]. Similar results were obtained from yellow inner leaves compared to white inner leaves (Table 1). Collectively, these data confirmed that carotenoid profiles of the yellow inner leaf cultivars of Chinese cabbage were obviously higher than and different from those of common white leaf cultivars [1]. However, there is no report on the involvement of related genes and its expression. We found out that expression of lutein and *β*-carotene biosynthesis-related genes (*BrPSY*,* BrPDS*,* BrRUT*,* BrHYDB*, and* BrCRTISO*) is high in yellow inner leaves, whereas expression of its degradation-related genes is low (Table 2). In the carotenoid biosynthetic pathway, phytoene synthase (PSY) catalyzes the first committed reaction of the head to head condensation of two geranylgeranyl diphosphate (GGPP) molecules [20]. BrHYDB acts as a catalyst for the production of zeaxanthin hydrolyzed from *β*-carotene [32–34].* BrPSY* and* BrHYDB* are believed to be responsible for *β*-carotene and zeaxanthin synthesis [20]. The primary role of BrCRTISO is as an enzyme that converts prolycopene into lycopene in* B. rapa* plants. In this study, comparative analysis with* Arabidopsis* counterparts revealed that* BrCRTISO* is likely responsible for carotenoid biosynthesis. These results are similar to those reported for* Arabidopsis*, which encodes a functional carotenoid isomerase and causes prolycopene accumulation when mutated [35]. Lee et al. [30] reported that* BrCRTISO1* was not normally expressed in orange cultivars and this result indicates that a lack of* BrCRTISO1* transcript was the cause of sequence variation in the orange cultivars when compared to the counterpart gene in the yellow leaves cultivars. This could be one of the causes responsible for the yellow inner leaves phenotype.

We identified 1 unknown gene and 9 putative genes responsible for yellow pigmentation (Table 3). These genes showed high expression in yellow leaves when compared with white leaves. Moreover, some stress-related genes-like such as chitinase, dynein light chain, and reductase genes [36] also showed high expression in yellow leaves (Table 3). Although these genes are not involved in carotenoid biosynthesis, this might be one of the causes of pigmentation. In addition, a large number of no_hit_found genes (novel genes in* B. rapa*) (180 genes) were specifically expressed in yellow leaves (Table S5). It will be essential for identifying function of these genes in relation to leaf color determination. Based on these findings, functional studies should be conducted to clarify the nature of these unknown genes in Chinese cabbage.

We identified 21 putative transcription factor genes associated with yellow color in* B. rapa*. Basic helix-loop-helix proteins and MYB proteins also function together to control flower pigmentation in snapdragon [37] and petunia [38]. MYB transcription factors act as the primary component of color differences between plant varieties such as potato, tomato, pepper [16], and grape [39] and in some species of* Antirrhinum* [40]. Zinc binding proteins are triggered via specific mechanisms related to transcription factors involved in important biological processes [41]. These facts may support that these TFs will participate in the regulation of carotenoid content in* B. rapa*.

Regarding the three transcription factors that were highly expressed in yellow inner leaves (Table 4, Table S9), none of them has been reported as gene as determining color formation.* Arabidopsis* orthologous (A20/AN1-like zinc finger protein) of* Brapa_ESTC013161* shows induced expression by stress with redox-dependent regulation [42], and* Arabidopsis* BIM1 controls embryonic patterning through brassinosteroid signaling [43]. There is no report on the function of* Brapa_ESTC006452* orthologous (*ZFP8*) yet. All these results suggest that transcription factors showing yellow-specific expression might be related to control the carotenoid biosynthesis in* B. rapa*.

In conclusion, we identified three genes as functionally novel genes that are involved in yellow color pigmentation and also a large number of unknown genes exhibit functionally diverse transcription factor activity, using newly developed 300K* Brassica rapa* microarrays. These unknown genes may be responsible for yellow pigmentation in Chinese cabbage and the results showed that biological functions of particular transcription factors may be activated in the pigmentation pathways. Despite identification of some stress related genes not related to carotenoid biosynthesis-like chitinase, reductase proteins might be one of the causes for the yellow color trait. Overall, the results presented herein will provide a tool for future transcriptome studies and investigation of genes involved in carotenoid biosynthesis of Chinese cabbage as well as valuable insight into the response to yellow pigmentation production.

## Supplementary Material

In this study, we used Br300K *B. rapa* microarray and identified three genes as functionally novel genes that involved in yellow color pigmentation. Supplementary figures (Figure S1 to S3) explains the information about plant materials and correlation coefficient of two experimental results. The three identified transcription factor genes are specifically expressed and confirmed their relatedness in yellow pigmentation. Table S1 to S9 supports our results and provides detailed annotation information of some differentially expressed genes.

## Figures and Tables

**Table 1:** Content of lutein and *β*-carotene in two contrasting Chinese cabbage cultivars.

*B. rapa* cultivar	Lutein (*μ*g/g dry weight)	*β*-carotene (*μ*g/g dry weight)
Kenshin (white inner leaves)	0.19 ± 0.08	0.69 ± 0.08
Wheessen (yellow inner leaves)	**47.49 **±** 1.59**	**12.00 **±** 0.61**

**Table 2:** Carotene and lutein synthesis-related genes.

Classification	Gene name	*Br* Chip ID	*At* locus	PI value		RT-PCR	
				White	Yellow	W	Y
Alpha-, beta-carotene synthesis	*BrPSY *	Brapa_ESTC045764	At5g17230	2,591	8,978	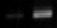	
	*BrPDS *	Brapa_ESTC002927	At4g14210	198	644	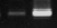	
	*BrCRTISO *	Brapa_ESTC016346	At1g06820	199	565	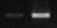	
	*BrRUT2 *	Brapa_ESTC013530	At5g57030	172	522	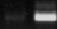	

Lutein and zeaxanthin synthesis	*BrRUT1 *	Brapa_ESTC043629	At3g53130	956	1,544	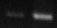	
	*BrRUT5 *	Brapa_ESTC000882	At1g31800	173	444	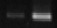	
	*BrHYDB1 *	Brapa_ESTC013493	At4g25700	1,995	2,768	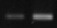	
	*BrHYDB2 *	Brapa_ESTC006182	At5g57030	1,044	4,856	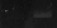	

Degradation	*BrNCED3 *	Brapa_ESTC007729	At3g14440	1,848	1,622	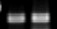	
	*BrNCED4 *	Brapa_ESTC026102	At4g19170	8,361	4,453	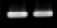	
	*BrNCED6 *	Brapa_ESTC022333	At3g24220	113	106	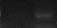	

Color	*BrPAC1 *	Brapa_ESTC007240	At2g48120	4,656	1,719	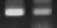	
	*BrOR *	Brapa_ESTC013943	At5g61670	901	702	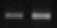	
	*BrIM *	Brapa_ESTC010363	At4g22260	1,285	1,523	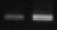	
	*BrACT1 *					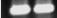	

**Table 3:** Genes expressed specifically in either yellow or white inner leaves.

Expression pattern	Gene name	*Br* SEQ ID	*At *locus	PI value		RT-PCR	
				White	Yellow	W	Y
Yellow specific	Unknown protein	Brapa_ESTC005061	At4g30660	37	11,124	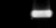	
	Chitinase	Brapa_ESTC005522	At3g47540	34	13,990	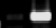	
	CA18	Brapa_ESTC013802	At5g14740	38	8,063	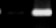	
	Dynein light chain	Brapa_ESTC010078	At4g27360	124	20,072	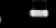	
	Unknown protein	Brapa_ESTC010121	At4g10300	24	3,243	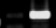	
	Reductase	Brapa_ESTC013704	At5g52100	462	18,255	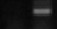	
	Calcium binding	Brapa_ESTC035004	At1g15860	497	11,041	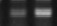	
	APX4	Brapa_ESTC013724	At4g09010	437	12,439	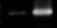	
	SHM2	Brapa_ESTC017490	At5g26780	466	4,033	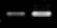	
	SBPase	Brapa_ESTC047526	At3g55800	440	7,571	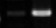	
	Thylakoid protein	Brapa_ESTC011620	At5g52970	456	6,459	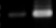	
	Unknown protein	Brapa_ESTC006516	At3g19800	458	4,961	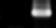	
		Brapa_ESTC048334	no_hits	13	21,498	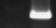	
		Brapa_ESTC042517	no_hits	16	25,294	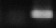	
		Brapa_ESTC028538	no_hits	32	16,831	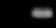	
		Brapa_ESTC006072	no_hits	13	4,401	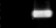	
		Brapa_ESTC000684	no_hits	91	28,921	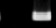	
		Brapa_ESTC041578	no_hits	86	17,882	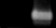	
		Brapa_ESTC002681	no_hits	175	29,165	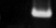	
		Brapa_ESTC002391	no_hits	197	27,114	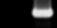	
		Brapa_ESTC048903	no_hits	403	15,662	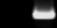	
		Brapa_ESTC002008	no_hits	444	19,448	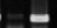	

White specific	Thaumatin protein	Brapa_ESTC024359	At4g36010	7,666	125	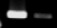	
		Brapa_ESTC002914	no_hits	5,441	62	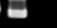	
		Brapa_ESTC048544	no_hits	2,635	27	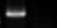	
		Brapa_ESTC028228	no_hits	1,891	21	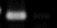	
	*BrACT1 *					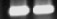	

**Table 4:** List of transcription factors expressed specifically in yellow inner leaves.

*B. rapa* SEQ_ID	*TAIR7_cds ID *	A_thaliana_homologue_Description	PI value		Fold Change (Y/W)
			White leaves	Yellow leaves	
Brapa_ESTC013161	AT1G51200	A20/AN1-like zinc finger protein	161	2674	16.6
Brapa_ESTC025847	AT5G08130	BIM1 (BES1-interacting Myc-like protein 1)	280	3517	12.5
Brapa_ESTC006452	AT2G41940	ZFP8 (ZINC FINGER PROTEIN 8)	269	3323	12.4
Brapa_ESTC024811	AT5G64810	WRKY51 (WRKY DNA-binding protein 51)	155	1002	6.5
Brapa_ESTC017689	AT1G51600	ZML2 (ZIM-LIKE 2)	472	3009	6.4
Brapa_ESTC017762	AT2G18328	DNA binding	401	2336	5.8
Brapa_ESTC004343	AT4G16420	ADA2B (PROPORZ1)	286	1593	5.6
Brapa_ESTC051029	AT1G49950	ATTRB1/TRB1 (TELOMERE REPEAT BINDING FACTOR 1)	396	1918	4.8
Brapa_ESTC047297	AT1G49950	ATTRB1/TRB1 (TELOMERE REPEAT BINDING FACTOR 1)	400	1902	4.8
Brapa_ESTC032962	AT4G31060	AP2 domain-containing transcription factor, putative	382	1582	4.1
Brapa_ESTC006124	AT5G64810	WRKY51 (WRKY DNA-binding protein 51)	312	1166	3.7
Brapa_ESTC001675	AT4G17810	Nucleic acid binding/transcription factor/zinc ion binding	442	1601	3.6
Brapa_ESTC011816	AT3G51960	bZIP family transcription factor	309	1079	3.5
Brapa_ESTC037364	AT4G12020	WRKY19 (WRKY DNA-binding protein 19)	412	1394	3.4
Brapa_ESTC014402	AT2G40750	WRKY54 (WRKY DNA-binding protein 54)	322	1021	3.2
Brapa_ESTC035091	AT3G57600	AP2 domain-containing transcription factor, putative	455	1411	3.1
Brapa_ESTC011257	AT4G34680	GATA transcription factor 3, putative (GATA-3)	371	1095	3.0
Brapa_ESTC025927	AT3G17609	HYH (HY5-HOMOLOG)	392	1152	2.9
Brapa_ESTC035196	AT4G30410	Transcription factor	480	1248	2.6
Brapa_ESTC025999	AT3G50890	ATHB28 (ARABIDOPSIS THALIANA HOMEOBOX PROTEIN 28)	464	1189	2.6
Brapa_ESTC030153	AT1G79180	AtMYB63 (myb domain protein 63)	470	1023	2.2
