# Supplementary material for: Identification of Yellow Pigmentation Genes in Brassica rapa ssp. pekinensis Using Br300 Microarray
Source: Int J Genomics. 2014 Dec 31;2014:204969. doi: 10.1155/2014/204969 (PMC4297637; doi:10.1155/2014/204969)
Supplement: Supplementary file 1 — In this study, we used Br300K B. rapa microarray and identified three genes as functionally novel genes that involved in yellow color pigmentation. Supplementary figures (Figure S1 to S3) explains the information about plant materials and correlation coefficient of two experimental results. The three identified transcription factor genes are specifically expressed and confirmed their relatedness in yellow pigmentation. Table S1 to S9 supports our results and provides detailed annotation information of some differentially expressed genes. [file 204969.f1.zip › description.docx]

In this study, we used Br300K *B. rapa* microarray and identified three genes as functionally novel genes that involved in yellow color pigmentation. Supplementary figures (Figure S1 to S3) explains the information about plant materials and correlation coefficient of two experimental results. The three identified transcription factor genes are specifically expressed and confirmed their relatedness in yellow pigmentation. Table S1 to S9 supports our results and provides detailed annotation information of some differentially expressed genes.

Additional file 1as **Figure S1** provides information of two different plant material used in this study.

Additional file 2 as **Figure S2** explains the correlation coefficient between replicated microarray analyses.

Additional file 3 as **Figure S3** explains the correlation coefficient between RT-PCR and microarray results.

Additional file 4 as **Table S1** provides the primer sequences used for RT-PCR experiments.

Additional file 5 as **Table S2** provides UniGene information of the 300K microarray chip.

Additional file 6 as **Table S3** provides detail annotation information of differentially expressed genes.

Additional file 7 as **Table S4** provides information of top 20 up-regulated genes.

Additional file 8 as **Table S5** provides information of specifically expressed genes in yellow leaves

Additional file 9 as **Table S6** showing yellow specific genes had PI values of over 1000.

Additional file 10 as **Table S7** showing white specific genes had PI values of over 1000.

Additional file 11 as **Table S8** provides detail annotation information of transcription factor genes.

Additional file as **Table S9** provides the carotenoid content of Arabidopsis Ko-mutant corresponding to transcription factor genes.
