# Supplementary material for: Identification of Yellow Pigmentation Genes in Brassica rapa ssp. pekinensis Using Br300 Microarray
Source: Int J Genomics. 2014 Dec 31;2014:204969. doi: 10.1155/2014/204969 (PMC4297637; doi:10.1155/2014/204969)
Supplement: Supplementary file 1 — In this study, we used Br300K B. rapa microarray and identified three genes as functionally novel genes that involved in yellow color pigmentation. Supplementary figures (Figure S1 to S3) explains the information about plant materials and correlation coefficient of two experimental results. The three identified transcription factor genes are specifically expressed and confirmed their relatedness in yellow pigmentation. Table S1 to S9 supports our results and provides detailed annotation information of some differentially expressed genes. [file 204969.f1.zip › mat.204969.v2/Table S1.doc]

**Table S1. Primers used for RT-PCR analysis and T-DNA mutant confirmation.**

| **Gene name** | **SEQ_ID** | **Primer Sequence** | |
| --- | --- | --- | --- |
|  |  | **Forward primer** | **Reverse primer** |
| ***BrPSY*** | Brapa_ESTC045764 | TTGATGATCTCTACCTTTACTGCTACTATGTAGC | CCTCTTAAGTTGTTTATCTTGAACTTGAAGC |
| ***BrPDS*** | Brapa_ESTC002927 | GAAACATGGATCGAAGATGGCG | TTTAGCCTTGCTTTGGTCAGCG |
| ***BrCRTISO*** | Brapa_ESTC016346 | TCCACATGGGTGTTAAAGCAGAGG | ATGGTCTTGAGCAACACTTCCACTAG |
| ***BrLCYE* (*BrRUT2*)** | Brapa_ESTC013530 | TAAAATGGAGTGTGTTGGTGCTCG | AATAACCACTAGGTCCAAAGCACCAC |
| ***BrCYP93C* (*BrRUT1*)** | Brapa_ESTC043629 | CGTCACTCACTCGCACCATCAC | TTCCCTCCACTGCATAAGGCTG |
| ***BrCYP97A3* (*BrRUT5*)** | Brapa_ESTC000882 | ATCGCAACCTGCAAGAGAATGG | TATGCATTTCCGAGGTCCTCCA |
| ***BrHYDB1*** | Brapa_ESTC013493 | CGGAGGCAGAGTTCTCCAATCA | AGGAACAGCGTTGACTATGGCG |
| ***BrHYDB2*** | Brapa_ESTC006182 | ACAAACCTCCCTTCTATGGCGG | AACGACGGCACCAATTGAGAAA |
| ***BrNCED3*** | Brapa_ESTC007729 | TCAAGCTCCAGGAGATGATCCG | TCCCTGCTTCGAGGTTGACTTG |
| ***BrNCED4*** | Brapa_ESTC026102 | GACACCATCGTTCTCATCGC | TCACCACGTAACCGTCATCC |
| ***BrNCED6*** | Brapa_ESTC022333 | GCTAGCAGCTTCCATGCTCGAC | CAAGCTCGGCTTCCTGAACAAG |
| ***BrPAC1*** | Brapa_ESTC007240 | ATGGCTTTGGAGACGATGCTTG | TTGACGGATGGTTCGCTTCTTC |
| ***BrOR*** | Brapa_ESTC013943 | TTTCACGGCCTTGGACTCTGAC | AGGATGATCCCAGCGATGAGTG |
| ***BrIM*** | Brapa_ESTC010363 | CTCTGGAGGCTTCGGTCATCAA | CAGGTGGAGGCGATTTCTTCAA |
| **No_hits** | Brapa_ESTC000684 | CGATTGCCTCTCGATCTGAA | AGACAAGGAAAGCACGGGAT |
| **No_hits** | Brapa_ESTC002008 | GTTCTCCGGTTACGGTCCAT | GTCAGCAGAACTGAAGCCGA |
| **No_hits** | Brapa_ESTC002391 | TGATCTCGTTGCCCGTGTAT | CCGACAAAGCTTCAATTCCA |
| **No_hits** | Brapa_ESTC002681 | TAAGTAGCCGGTATGGCGTG | CCCGGAATACACTTTAGCCC |
| **No_hits** | Brapa_ESTC002914 | GACATTTTGGTGTCCTTGCG | CCGGATCACCCCATTTATTT |
| **Unknown protein** | Brapa_ESTC005061 | AGTGCGAAATCTTGTGCGAG | CCGATGTATCAAAACACACAATG |
| **Chitinase** | Brapa_ESTC005522 | GGATGTGCCGGAAAAAGATT | CACCGTTACATTCGATTCCG |
| **No_hits** | Brapa_ESTC006072 | GGGTTTAGTTTGGGGGTTTG | TTGTAGGAATAATAAGGGAAACGA |
| **Unknown protein** | Brapa_ESTC006516 | TTCTTCTTCTTCGGCAGCAA | GCTGAAGCTCGATTCTCCCT |
| **Dynein light chain** | Brapa_ESTC010078 | GGTCAGCAGTGATGGGTGAG | CAAACCGGAACAACGATTGA |
| **Unknown protein** | Brapa_ESTC010121 | TCCACCCGAGTCCACACTAA | TCGAAGCAAACAAATTCCAAA |
| **Thylakoid protein** | Brapa_ESTC011620 | CGGAGCTCTAGCTCTCGGTT | CATGTTGCCGTATTTCCCAG |
| **Reductase** | Brapa_ESTC013704 | CTAAAGCCAGGGGAATGGAG | GGTGGCCTTGTCACAAAGTG |
| ***BrAPX4*** | Brapa_ESTC013724 | TCTCAAACTCGCTGGGACTG | AGGTCCACCCTTTGAGAACG |
| ***BrCA18*** | Brapa_ESTC013802 | CCGACTGGTATCAGCGAAAA | AAATTTAGCACGTGCGATGG |
| **Thaumatin protein** | Brapa_ESTC024359 | GACGCCGGAGTATTGCTGTA | AGCAACTTTGGAATCCCCAC |
| **No_hits** | Brapa_ESTC028228 | TGGATTTTGCGGTAGTGGTG | AGCTTCCGGTTTATGGGATT |
| **No_hits** | Brapa_ESTC028538 | TTGCGGCGAGATACCTCTTT | GGAGATTCATGATCGAGAGGC |
| **Calcium binding** | Brapa_ESTC035004 | ACAAGAGGCCTTGTGGCTTT | GTGCAGTCGTATCCTCACCG |
| **No_hits** | Brapa_ESTC041578 | CCGGTGGATTGGGTTTAGTT | GCACCAGGTCCAGTTCTGTG |
| **No_hits** | Brapa_ESTC042517 | GAACAAATTCAGCTCCGCAA | GGTTTGCATGCCTTCTTCAA |
| **No_hits** | Brapa_ESTC045302 | TTAAGGTCAGGAAGGCCGAT | ACACCTCGGTTGCTTCCTTT |
| **No_hits** | Brapa_ESTC048334 | TCTTGCCGTTTCAACCTCAG | CGCAAAGAGTCGGAACAAAA |
| **No_hits** | Brapa_ESTC048544 | TGGCTGCTCAGTTGGAAAAG | AGAGATGTGGCTGGTGGTGA |
| **No_hits** | Brapa_ESTC048903 | GGCTTATGTTTTTCGGGAGG | CACCATGTTTTCCAACGGAG |
| ***A20/AN1*-like** | Brapa_ESTC013161 | CGTACGAAATGGACCACGAC | CCACAGCGACATTTGAATCC |
| ***BrBIM1*** | Brapa_ESTC025847 | ATTCTGCAACAGAGCAACGG | CACATTCCATTGCAGCAACA |
| ***BrZFP8*** | Brapa_ESTC006452 | ATAACTCCTCCGGCACTGCT | CTAGCTTCCGTTGTCCACGA |
| ***BrWRKY48*** | Brapa_ESTC024831 | CGTTGCACCACAGTAGGTTGT | AATGAAAGTACCATCGGGGG |
| ***BrWRKY11*** | Brapa_ESTC024467 | CCCCGAACATAACAACAACG | ACGCGAGGAAGATCGAAGAT |
| ***BrANAC055*** | Brapa_ESTC012118 | CGCTATTCGGGGAAAAAGAC | AACATCCTTTTGTGCGCTTG |
| ***BrCCA1*** | Brapa_ESTC008890 | CTCCGACTAACCAGGAAGCC | CGATCTTTTGCCAGGCTCTA |
| ***BrABR1*** | Brapa_ESTC009801 | AGCCGACCACTCGAGAAGAT | TATGTTGGGCCTGAACCTGA |
| ***BrMYB*** | Brapa_ESTC018840 | CAGAAATTTCGGCAAGGGAG | TCCCTAGCTGTCTCAAGCCC |
| ***BrHB6*** | Brapa_ESTC020445 | GGGAATGGGGAAAACAAAGA | GGTTTGTCCTCTTTCCTCGG |
| ***BrBZIP53*** | Brapa_ESTC038316 | ACGGACGAGAGGAAGAGGAA | GAAGCCCTTATCGGTTGCAT |
| ***BrSTO*** | Brapa_ESTC010997 | CAAGTTCCCTCGTTGCGATA | TGTGAAACGGAAAGCTCAGG |
| ***BrACT1*** | Brapa_ESTC015555 | AGATCGTCCCCGGCTTCAAA | CAAGGCGTAAACGACGCAGG |
| **SALK_045674** | | TCCTATGCTTCCATACGCTAA | GGGCTTGAGAAGTTGGGAAGT |
| **SALK008677C** | | CTTCCCTCAACAACTATGTTAC | TCCAACACTACCTCTCTCCACAG |
| **SALK_098442C** | | TGCGGTTAGCATAACCGGGT | GAAGTTCTTGAGGGTAACATCAG |
| **SALK_012835** | | AGAAGCAGATTCATCCCACAG | TGGTACGTGAAGCAGGTAGTCAC |
| **T-DNA left border** | | GTGGACCGCTTGCTGCAA |  |
